# Transobturator tape material detected in the bladder neck: a case report

**DOI:** 10.1186/s13256-019-2059-y

**Published:** 2019-04-30

**Authors:** Yavuz Güler, Akif Erbin, Burak Üçpınar

**Affiliations:** 1 Department of Urology, Safa Hospital, Istanbul, TR Turkey; 2 Department of Urology, Haseki Traning and Research Hospital, Istanbul, Turkey

**Keywords:** Transobturator tape surgery, Stress incontinence, Neuro-urology, Uro-gynecology

## Abstract

**Introduction:**

Stress urinary incontinence surgeries (transobturator tape and tension-free vaginal tape) are safely performed with success rates over 90%. The transobturator tape procedure attracted more attention due to the lack of major complications, such as intraabdominal organ and vascular injuries, related to the tension-free vaginal tape procedure. Although there are no major or mortality-related complications, more lower urinary complaints, especially vaginal erosion, are reported in transobturator tape surgery. Here we present a rare complication of transobturator tape surgery: the accidental placement of mesh material in the bladder neck. With this case report, we aimed to discuss the diagnosis and management of misplaced transobturator tape material.

**Case presentation:**

A 38-year-old Caucasian woman who had stress urinary incontinence that had persisted for 6 years underwent transobturator tape surgery in a different clinic 2 years ago. Subsequently, she presented to our clinic with lower urinary tract complaints such as incontinence and dysuria. A physical examination was unremarkable besides total incontinence. A diagnostic cystoscopy was performed and sling material that crossed her bladder neck from 3 o’clock to 10 o’clock was identified. The misplaced transobturator tape material was cut endoscopically with an internal urethrotomy knife. Afterwards, a midurethral incision was made and mesh parts were removed bilaterally. After successful removal of the mesh material, a new transobturator tape was placed.

**Conclusions:**

Even though transobturator tape surgery is a safe and effective procedure for stress urinary incontinence, certain complications can be encountered. Misplacement of the mesh material through the bladder neck is a rare complication and can be managed by successfully removing the mesh material and appropriately placing new transobturator tape material.

## Background

Midurethral vaginal suspension surgeries are successful surgical methods that provide patient satisfaction rates of over 90% for stress urinary incontinence (SUI) [1, 2]. Tension-free vaginal tape (TVT) was introduced in 1995 by Ulmsten *et al*. [3]. In spite of its high success rates, undesired bowel and bladder injuries were observed [4, 5]. To reduce these complications, Delorme described the transobturator midurethral route in 2001 [6]. However, bladder and urethral injuries were also observed in the transobturator tape (TOT) procedure, even though the retropubic route was not used [7–10]. Estimating the incidence and prevalence of midurethral sling-related complications and quickly recognizing and managing them properly are key factors in improving quality of care of patients who undergo surgery for SUI. In this case report, we aimed to discuss the diagnosis and management of misplaced TOT material.

## Case presentation

A 38-year-old Caucasian woman with a history of TOT surgery 2 years ago presented to our hospital with complaints of urinary incontinence that emerged during coughing, walking, and physical exercises or activities. She had also experienced dysuria and urine leakage during sexual intercourse. Therefore, she had not had regular sexual intercourse for 2 years. Prior to her admission to our hospital, she was diagnosed as having SUI and used duloxetine (80 mg, daily) for 3 months. She took no other medications on a regular basis. She had performed Kegel exercises routinely. However, her symptoms persisted. She was a housewife, with no history of alcohol consumption or tobacco smoking. She had had two deliveries: one vaginal birth and one cesarean delivery. There was no similar history of illness in her family.

**Fig. 1:**
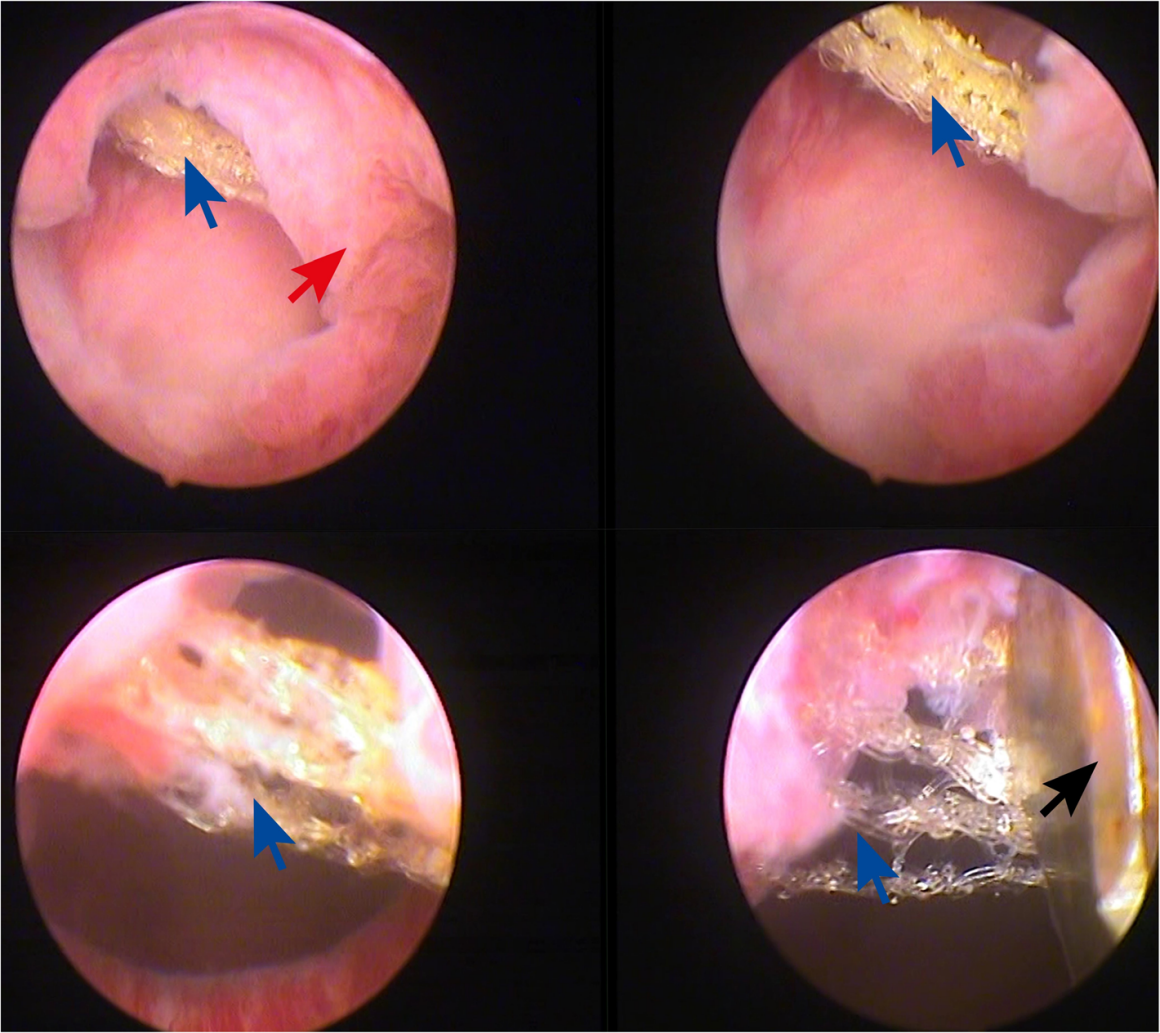
The *blue arrows* show the mesh material passing through the bladder neck. The *red arrow* indicates the granulation tissue around the sling material. The *dark arrow* shows the urethrotomy knife

On admission, her temperature was 36.6 °C, pulse was 82 beats/minute, and blood pressure was 110/65 mmHg. She was fully conscious and responsive. Psychologically, she was depressed. On systematic physical examination, no abdominal tenderness and no anatomic anomalies were detected. No murmurs or arrhythmia were detected during auscultation of her heart. Respiratory frequency was 14/minute and no wheezing or rales were detected. On neurological examination, her muscle strength and tone were normal. Ulnar, patellar, and Achilles reflexes were all normal (2+). A urogynecological physical examination revealed SUI without any urogenital prolapse. In laboratory analysis, her total white blood cell count was 6.9 × 103/mm^3^, hemoglobin was 12.1 g/dL, alanine aminotransferase was 38 u/l, aspartate aminotransferase was 35 u/l, C-reactive protein was 1.1 mg/l, creatinine was 0.6 mg/dl, and serological tests were negative: hepatitis B surface antigen (HbsAg), anti-hepatitis C virus (HCV), and anti-HIV. Urine analysis showed microscopic hematuria and urine culture was sterile. Post-void residual volume was insignificant. A diagnostic cystoscopy was performed and sling material which crossed her bladder neck from 3 o’clock to 10 o’clock was identified (Fig. 1). The mesh material was cut with an endoscopic internal urethrotomy knife and retrieved by using foreign body grasping forceps. Other mesh parts were excised through a transvaginal midurethral incision. New TOT material was placed and the procedure was terminated. Our patient was discharged on the first postoperative day. First week, 3-month, and 6-month follow-up visits showed complete absence of urinary incontinence and other urinary complaints.

## Discussion

We present a case who underwent a TOT procedure 2 years ago and who we diagnosed as having sling material in her bladder neck. She had persistent dysuria and total incontinence, besides multiple unsuccessful treatments. At a diagnostic cystoscopy, the misplaced sling material was identified. After successful removal of the mesh, new TOT material was placed in the same session.

The success rates of transvaginal sling operations, whichever is applied, are above 90%. In addition, patients with SUI are treated with sling surgeries that offer high success rates with minimally invasive surgeries and rapid recovery rates. The main measure of success is vaginal dryness and patient satisfaction [1, 2, 11, 12]. In 1995, Ulmsten *et al*. first introduced the TVT method [3]. However, in 2001, the transobturator route was described by Delorme to reduce major complications, such as bladder and bowel injuries, related to the TVT procedure [6]. Although fewer complications have been reported for TOT procedures than for TVT procedures, complications such as bladder perforation have been reported for TOT procedures [7–10]. Since 2011, mini-sling methods have been applied to reduce complication rates [13].

Misplacement of the mesh material inside the bladder is rarely encountered in sling surgeries. The incidence of sling material placement in the bladder and urinary tract is reported to be between 1 and 6% in the literature. However, during TOT procedures, bladder perforation is less common, because the retroperitoneal route is not used [1, 2, 4, 14–19]. Perforation of the bladder or urethra may occur during abrupt dissection with scissors, incorrect placement of the trocar, passage of the needle through the bladder or urethra, or erosion of the urethra or bladder due to the suspension material [2]. Our patient had undergone TOT surgery in a different hospital 2 years ago and details of the operation were not known. We thought that these findings might be due to urethral erosion or wrong trocar access. During the operation, if entry of the trocar into the bladder is noticed, the path of the needle can be corrected and the sling material can be re-placed into the suburethral space. In other words, this inaccuracy can be corrected intraoperatively, without further complications [6].

Predisposing factors for these complications can be listed as surgeons’ lack of experience, presence of cystocele, advanced patient age, history of tobacco smoking, presence of diabetes mellitus, patients with low body mass index (BMI), and patients with a history of vaginal or pelvic surgery [20, 21]. Cystoscopy is recommended for cases with predisposing factors for complication or suspected perforation. However, it is not always possible to identify the misplaced material, if it is very closely related to the bladder neck. Overlooked cases despite cystoscopy have been reported [6, 20]. During cystoscopy, the use of 70-degree lenses is particularly important to visualize the bladder neck and avoid misdiagnosis. In patients with bladder perforations, attempts are made to medically treat complaints, such as urinary incontinence, increased urinary frequency, dysuria, recurrent urinary tract infection, urgency, incontinence, hematuria, and vaginal discomfort; therefore, it can take months or even years to diagnose and treat the underlying pathology [20, 22]. The average duration between diagnosis and treatment is 7–36 months [5, 20, 22].

Most of the patients who have undergone midurethral sling surgery develop calcification and a stone in the suspension material [5, 23]. For patients who have undergone midurethral sling surgery and have had lower urinary tract complaints with or without bladder stone detection in the postoperative period, the possibility of bladder and urethral perforation should be kept in mind. Diagnostic cystoscopy is crucial to confirm the diagnosis in patients with suspected perforation. Various treatment modalities can be used for the treatment of misplaced mesh material. Endoscopic knife, transurethral electrocautery, holmium: yttrium-aluminum-garnet (Ho: YAG) laser, laparoscopy combined with endoscopy, and open cystotomy can be listed as described treatment alternatives [5, 18, 22, 23].

Jo *et al.* reported 23 bladder and urethral perforation cases in patients who had undergone TOT (*n* = 11) and TVT and mini-sling (*n* = 12) surgeries [22]. Among these patients, 15 of them were treated by transurethral resection-electrocautery (TUR-E) and 8 of them were treated by transurethral resection-holmium (TUR-H) surgery. They have also performed midurethral vaginal incision for the removal of remaining suspension parts [22]. There are various other treatment options for the removal of inappropriately placed mesh material. Chan and Tse shared a bladder perforation case in a 71-year-old woman who had undergone intravaginal slingplasty (IVS) surgery 9 months ago [18]. She presented with lower urinary tract symptoms and sling material was detected inside her bladder during a cystoscopic examination. A laparoscopic approach was preferred for the removal of the sling material. Ozdemir *et al.* preferred open cystolithotomy and Burch surgery for a 56-year-old patient with bladder perforation due to a prior TVT operation [5]. Foley *et al*. removed the sling material by cutting it through the urethral meatus with a scissor by using a nasal speculum [23]. Recognizing and treating bladder perforation can relieve lower urinary tract symptoms like dysuria and urgency, however, SUI should be treated effectively, which was the underlying reason for surgery in the beginning. Foley *et al.* reported that re-placement of a new mesh material is effective in treating SUI and those who did not accept new sling material had persistent symptoms after perforation surgery [23]. In our patient, after we removed the previously placed sling material, we placed new TOT sling material and we ensured the continence of our patient.

## Conclusion

Even though TOT surgery is a safe and effective procedure for SUI, complications can be encountered. Misplacement of the mesh material through the bladder neck is a rare complication and can be managed by successfully removing the mesh material and appropriately placing new TOT material. Cystoscopic evaluation is not recommended routinely, but it must be performed if the patient has sufficient complications to create doubt.

## Abbreviations

SUI: Stress urinary incontinence
TOT: Transobturator tape
TVT: Tension-free vaginal tape
